# Barriers and facilitators to hospital pharmacists’ engagement in medication safety activities: a qualitative study using the theoretical domains framework

**DOI:** 10.1186/s40545-018-0129-y

**Published:** 2018-01-23

**Authors:** Alemayehu B. Mekonnen, Andrew J. McLachlan, Jo-anne E. Brien, Desalew Mekonnen, Zenahebezu Abay

**Affiliations:** 1 Faculty of Pharmacy, University of Sydney, Pharmacy and Bank building (A15), Sydney, NSW 2006 Australia; 2 School of Pharmacy, University of Gondar, Gondar, Ethiopia; 3 Department of Internal Medicine, Addis Ababa University, Addis Ababa, Ethiopia; 4 Department of Internal Medicine, University of Gondar, Gondar, Ethiopia

**Keywords:** Medication safety, Patient safety, Medication-related problems, Pharmacists, Ethiopia

## Abstract

**Background:**

Hospital pharmacists play a central role in medication safety activities. However, in Ethiopia, this role has been launched recently and little is known regarding the current status of this extended service. Using the Theoretical Domains Framework (TDF), we aimed to identify the barriers and facilitators to hospital pharmacists’ engagement in medication safety activities across various public hospitals in the Amhara region of Ethiopia.

**Methods:**

Eight focus group discussions, using an interview guide that was drawn upon the TDF, were conducted with 44 hospital pharmacists to explore their beliefs regarding their involvement in clinical services. Group discussions were audio-recorded, transcribed verbatim, and analysed using directed content analysis based on the TDF. Relevant domains were identified by applying relevance criteria to each of the domains in the TDF.

**Results:**

Content analysis revealed six domains that influence hospital pharmacists’ engagement in medication safety activities. These domains included ‘Knowledge’, ‘Skills’, ‘Environmental context and resources’, ‘Motivations and goals’, ‘Social influences’ and ‘Social/professional role’. Most hospital pharmacists believed knowledge gap was an issue, as was the lack of training and supportive skills although some expressed as they were competent enough for their skills in identifying medication related problems. Most participants were very much enthusiastic for their extended roles and were positive towards the future of the profession; however, competing priorities along with the lack of remuneration and awareness (of other health care professionals) regarding the profession’s role were barriers to service delivery. There were also a number of resource constraints, such as staffing, infrastructure and government funding, and acceptance rate of pharmacist’s recommendation that were likely to influence the clinical practice of pharmacists.

**Conclusion:**

Using the TDF**,** this study identified a wide range of barriers and facilitators to hospital pharmacists’ engagement in medication safety activities in resource-limited settings. There existed considerable interrelationships between domains that were perceived to influence hospital pharmacists’ behaviours, and this may assist in designing behaviour change interventions that target common behavioural domains.

**Electronic supplementary material:**

The online version of this article (10.1186/s40545-018-0129-y) contains supplementary material, which is available to authorized users.

## Background

Medications are the most common health care interventions used to improve the health outcome of patients when used safely and appropriately. However, they are also the major source of patient safety incidents [1]. The issue of medication safety has received increased attention since the publication of *To Err is Human: Building a Safer Health System* [1] in the USA, and is now a concern of many other countries [2–4]. In developing countries such as Africa, patient harm from adverse events is thought to be higher than elsewhere the world [5, 6]. Also, medication errors and adverse drug events in Ethiopia are believed to be significant public health problems [7–11], and studies in this regard are increasingly been published.

Many studies have identified various strategies to improve medication safety in the hospital environment, including but not limited to, computerized physician order entry with or without clinical decision support [12–14], barcode technology [15], educational sessions [16], and pharmacist involvement [17–19]. Specifically, the role of the hospital pharmacist has been rapidly evolving beyond the traditional roles of medication dispensing and distribution to expanded clinical services [20], and their role in improving medication safety is well acknowledged. The hospital pharmacist plays a prominent role in cutting adverse drug events, and medication errors [20], and medication safety activities, such as drug use evaluation, admission medication histories, adverse drug reaction management, and participation in medical rounds are believed to be associated with reduced mortality rates [21]. Also, our previous systematic reviews confirmed the positive impact of pharmacists, particularly when pharmacists are engaged in medication reconciliation at care transitions [22, 23]. Unlike the developed countries, pharmacists’ involvement in direct patient care is a recent journey in Ethiopia [24]. Major changes in the curricula have been made after a 5-year Bachelor of Pharmacy (BPharm) with a 1-year clerkship program has been launched in 2009. To date, standards and guidelines have been endorsed nationally—for example, the *Ethiopian Hospital Reform Implementation Guidelines* that require pharmacists to deliver direct patient care services, and this is taken as a minimum regulatory standard in the health facilities by the Ethiopian Standards Authority and the Ethiopian Food, Medicine and Health Care Administration and Control Authority (FMHACA) [25]. However, little is known about the current status of the implementation of these extended services, as well as the barriers and facilitators experienced by hospital pharmacists in delivering patient care services in Ethiopian public health facilities. The present study was part of a larger project aimed at implementing pharmacist-led medication safety programs (i.e., medication reconciliation) [26], and the implementation of this service was guided by a theoretical framework to help identify the barriers and facilitators to hospital pharmacists’ engagement in medication safety activities in selected public hospitals in the Amhara region, in Ethiopia.

## Method

### Study setting and participants

The study was conducted in eight public hospitals, all located in the Amhara region in the north western part of Ethiopia*.* This region is inhabited by approximately 20 million people and comprised of 19 public hospitals, and 796 health centers [27]. The Ethiopian health care system is challenged by poor health care financing, and close to 80% of the health expenditure is dependent on out-of-pocket expense [28], and the population mainly receives health services from public health institutions.

Pharmacists were recruited from four teaching/referral and four district hospitals, and there were a total of 252 pharmacy staffs (pharmacists, 140; pharmacy technicians, 112) working in the studied hospitals at the time of data collection. Of the 140 hospital pharmacists, only 61 were involved in direct patient care or clinical pharmacy services and were eligible to be included in this study; that is, these pharmacists were either clinical pharmacists or graduate pharmacists of the new patient-oriented curriculum or pharmacists with an in-service training on clinical pharmacy services. The study was conducted between February and August 2016.

### Study design

This is a qualitative study using focus group discussions (FGD). FGDs were employed in this study because the interactive nature of focus groups is specifically important when group norms and cultural values of particular groups are of interest, and to explore the degree of consensus on a given topic [29], including implementation of an intervention to improve medication safety. Many factors can affect the adaptability of an evidence based intervention, and the success of implementation efforts depends on a careful assessment of barriers to, and facilitators of, the behaviour to be changed [30]. A theory based identification of such factors provides a theoretically robust evidence base to inform implementation of an intervention [30]. The underpinning theoretical model used in this study is the Theoretical Domains Framework (TDF).

Increasing the uptake of evidence into clinical practice and improving patient outcomes needs behaviour change. The TDF from health psychology provides the basis for such an approach, ensuring that a wide range of possible theoretical explanations for the behaviours can be considered. Built from 33 behavioural theories, the TDF was developed to make theories more accessible for implementation researchers [31]. According to Michie et al. [31], the TDF has 12 domains to explain behaviour change: (1) ‘Knowledge’, (2) ‘Skills’, (3) ‘Social/professional role and identity’, (4) ‘Beliefs about capabilities’, (5) ‘Beliefs about consequences’, (6) ‘Motivation and goals’, (7) ‘Memory, attention and decision processes’, (8) ‘Environmental context and resources’, (9) ‘Social influences’, (10) ‘Emotion regulation’, (11) ‘Behavioural regulation’ and (12) ‘Nature of the behaviour’. The TDF has been extensively applied across a range of clinical behaviours such as prescribing, adverse drug event reporting, and transfusion behaviours [32–35]. Similarly, the present study used the TDF to develop a theory informed intervention to understand the perceived barriers and facilitators to hospital pharmacist’s role in medication safety.

### Data collection

In this study, FGDs were guided by questions designed based on the TDF (Additional file 1). For each of the 12 domains that could act as facilitators or barriers to current medication safety practices, the authors developed several interview questions. The number of interview questions ranged between 2 and 5 for each of the 12 domains, for a total of 43 questions to cover a wide range of beliefs assigned to each domain. The questions were initially drafted by one researcher (ABM) and then refined by health service researchers (AJM, JEB) and discussed by the research team to check for clinical relevance. Interview guides were translated from English versions to the local language (Amharic) by two non-official translators who are native speakers and working in the health care industry and validated by two of the research group (ABM, DM).

Initially, pharmacists were selected using a purposive sampling strategy, and this was further facilitated with snowball sampling. Selection of participants also considered variations in health service structure (teaching/referral and district) to capture a wide range of beliefs in the clinical practice of pharmacists. Opportunistically, we also interviewed a mix of hospital pharmacists who were attending an in-service training from various public hospitals in the region. Participants were recruited by letter invitation, and those willing to participate were contacted after a signed consent form had been submitted. The principal investigator (ABM) conducted and led the FGDs using the translated version (Amharic) of the topic guide. Prompts were used when necessary and pharmacists were encouraged to talk about their internal beliefs and attitudes that may hinder them from providing clinical pharmacy services, including medication safety roles. The discussions approximately lasted between 60 to 90 min, and data were collected until a point of saturation was reached. All discussion sessions were audiotaped and recorded.

### Data analysis

An Amharic/English speaking investigator (ABM) carried out verbatim Amharic transcriptions of all interviews and then translated into English. A coding guide was prepared based on previously published definitions [31, 36] and utilized for the purpose of consistent reporting (Additional file 2). Using the 12 domains of the TDF as a coding framework, directed content analysis of texts into the theoretical domains was performed [37]. Briefly, the analysis involved identifying contextualized brief statements related to the barriers and facilitators to medication safety activities, categorizing statements into TDF domains and mapping the underlying theoretical constructs within domains. The theoretical domains that were judged to be relevant were identified by considering the frequencies of the beliefs reported, the presence of conflicting beliefs, and evidence of strong beliefs that may influence the behaviour under investigation [34]. In establishing domain relevance, all of these factors were considered concurrently. Conventional content analysis was also conducted, and both analyses approaches were employed so as not to miss any themes [35].

## Results

Forty-four hospital pharmacists took part in eight focus groups, comprising four to nine participants per group (Table 1). Participants represented from eight hospitals, the majority of whom were males (*n* = 39). The mean clinical experience and age of the participants were 2.4 and 25.8 years, respectively (Table 1).

**Table 1:** Number and characteristics of participants in each of the eight focus groups

Focus groups	Number of participants	Age, mean	Male, n	Experience (years), mean
DMH – FG1	5	24.6	5	2.0
DMH – FG2	5	24.8	4	1.8
FHRH – FG1	5	26.8	5	2.8
FHRH – FG2	5	27.2	4	3.0
GUH	6	26.5	5	2.4
DTH	9	26.0	7	2.6
FH	4	25.5	4	2.0
Mixed hospitals^a^	5	24.8	5	2.8
Total	44	25.8	39	2.4

^a^Hospital pharmacists from Metema, Woldiya, Gondar University, and Enat hospitals were involved. *Abbreviations:* *DMH* Debre Markos hospital, *FHRH* Felege Hiwot referral hospital, *GUH* Gondar university hospital, *DTH* Debre Tabor hospital, *FH* Finoteselam hospital

### Barriers and facilitators

Conventional content analysis across all focus groups did not reveal different themes, and thus, we present our findings according to our primary data analysis plan. Using the directed content analysis, barriers and facilitators perceived by hospital pharmacists as being more relevant to the delivery of medication safety activities were categorized within six of the TDF domains. These domains included ‘Knowledge’, ‘Skills’, ‘Environmental context and resources’, ‘Motivation and goals’, ‘Social influences’, and ‘Social/professional role’.

#### Knowledge and skills

In most of the discussions, participants did not distinguish between knowledge and skill domains—for example, participants mentioned the lack of knowledge and skills altogether as barriers to their activities, and thus, in this study, they are presented together.

Participants expressed mixed views regarding the level of knowledge and skill necessary for complete delivery of clinical services and most believed there was a lack of awareness for those pharmacists’ extended roles. To the extreme, awareness issues from the pharmacy side were severe and its implication in the service delivery process was highly significant. Because these were not usually supported with further training, most participants held a strong firm in that pharmacists who lacked the know-how about clinical services had greatly impacted the service delivery and believed they should be targets for future interventions.

*“…those [pharmacists] who have knowledge about the service, and know what the service is about, support the service we are doing. Whereas those who pass most of their time at dispensing and not have enough knowledge and awareness about clinical pharmacy are not considering as we are working” [Referral hospital, Focus group*#*2].*

Trainings were arranged occasionally; however, most were not suitable to the interest of strengthening clinical pharmacy services. “*Even the trainings are more focused on system strengthening like APTS* [Auditable Pharmaceutical Transactions and Services] *and they are so much science oriented. They are not clinical based” [Referral hospital, Focus group#3].*

It was mentioned that, initially, there were some kinds of in-service trainings organized for clinical pharmacists to equip them with communication skills and pharmaceutical care. But, this had been stopped for a while.

*“The training that was prepared for the generic pharmacists to equip them clinical knowledge was already stopped” [Referral hospital, Focus group#4].*

Participants also raised issues such as the lack of an evidence and guidelines that showed how much their input affects the clinical practice, and this was further supported by the lack of consistent service although hospital pharmacists were confident enough in their skills in identifying medication-related problems. For instance, medication review was done with a limited scope, and there were no organized ways to perform medication reconciliation. During ward visit, hospital pharmacists took medication history and used it for pharmaceutical care decisions; however, this was done inconsistently and the evidence-base was not clear to many.

*“…the history is important for our decision, and we are working on medication reconciliation and review although it is not uniform… This is what we are currently doing, but I am not quite sure how strong the evidence for this, probably we will going to evaluate in the process” [Referral hospital, Focus group#3].*

#### Environmental context and resources

Initially, this domain was found to be less relevant from the perspective of behavioural change theory. But, later we understood that this domain had significant interactions with hospital pharmacists’ viewpoints expressed in the other domains considered as relevant in this study, such as motivation and goals, social influences and social/professional role. Overall, environmental constraints were highly referred by hospital pharmacists as being a major barrier to the delivery of medication safety activities. In this part of the domain, there was none who mentioned enabling factors regarding resource issues and all shared a common reflection on the consequences of environmental constraints on their role. Ranges of resource constraints were raised as barriers. Unlike other clinicians, for example, there had not been any room available for practicing pharmacists nearby to the wards they were working.

*“We are going far from the place where we are, but other HCPs follows patients at their own site” [District hospital*, *Focus group#6].*

In contrast to dispensing role, hospital pharmacists perceived clinical services add substantial time commitments and associated with many hardships.

*“Even we are busy of reading at home, it is not different from an academic life” [Teaching hospital, Focus group#5]*.

*“Pharmacists don’t want to face hardships” [District hospital, Focus group#1].*

The majority of participants also stressed that the lack of human resource was the challenge for delivering clinical services. In the studied hospitals, staff attrition was common and most participants believed this had been increased recently. Unlike teaching and referral hospitals, district hospitals also faced a severe shortage of other resources, such as reference books, guidelines, and computers with internet access.

Participants reported that ward-based hospital pharmacy services were limited in scope and delivered inconsistently. For example, these services were not done over the weakened and duty programs were stopped for a while, and participants believed this had imposed work burden when getting back to work on Monday. Participants also felt that, if many of their concerns had been solved, they believed this may boost their energy and perceived how much the concerned bodies were ready to accept hospital pharmacists’ extended roles. However, most pharmacists hesitated whether this had been met, given the lack of government funding and support for these services. Although part of the problem was explained by budget deficits nationally, participants cited that at least the government can play a major role in the technical support of these extended services. Additionally, clinical services were rarely and irregularly documented though there were institutional variations. In most of the studied hospitals, pharmacy own documents prepared for the purpose of recording clinical activities were not part of the medical record, or if it had been in place, pharmacist’s documentation was done infrequently.

*“…we do believe there is a severe problem of clinical pharmacy documentation. There is no body who support us in this regard” [Referral hospital, Focus group#3].*

#### Motivation and goals

A range of conflicting views regarding hospital pharmacists’ motivation and goals were collected. For example, most participants believed that what they were doing was a mere initiative from their side and not a cascaded role that was approved and endorsed by the government. “*Now, most of us are doing this work because we are interested in this” [District hospital, Focus group#6].* However, creating something out of nothing was challenging, and lacked remuneration, and a concern among the majority of hospital pharmacists. Some participants stated that patients were highly benefited from the clinical services hospital pharmacists were giving although they themselves did not have any extra benefit for these additional clinical services.

*“From the perspective of staff, I am feeling like a person giving free service”* [*Referral hospital, Focus group#1].*

Hospital pharmacists urged concerned bodies in support of these services through a remuneration scheme, and they believed this would likely bring major changes in the clinical practice of pharmacists.

*“…as you most satisfied with these* [staff benefits]*, you will going to do more interventions, and these can bring a good outcome” [Referral hospital, Focus group#4].*

Although participants strongly believed that there should have been a complete provision of clinical services, these were not done because hospital pharmacists would like to prefer a less challenging job or else, as a result of human resource shortages, they had been placed for other hospital services such as dispensing roles.

*“As any human, they* [hospital pharmacists] *might prefer a less challenging job” [Referral hospital, Focus group#4].*

Participants also believed that, as a result of the cancellation of weakened and duty programs which were practiced before, staffs thought that this was the least incentive they were thinking of, and this had affected their moral negatively.

*“It is not fair to cancel the Saturday and Sunday services. Before, we did weekend services, and even there was duty program and we did CP service and those things at least moralize us” [Referral hospital, Focus group#1].*

Many participants emphasized why hospital pharmacists lacked the inspiration for delivering clinical services, whereas they mentioned that the curriculum is very much patient oriented unlike the previous courses, yet there were few hospital pharmacists struggled into the duty of dispensing with the mere reason of collecting an additional benefit from the extra hours, but this was not arranged for clinical services. And because of this, most pharmacists preferred dispensing to clinical services.

*“We suffered so much when we studied CP and the work is challenging, but we are treated as previous pharmacists who studied a little bit advanced courses. There are many challenges with us. There are many differences in the curriculum, but there are things you will lose. For example, the region allows duty only for dispensing, and for this reason, at least to collect 500 or 600 birr for the duty program we are doing it rather than the clinical service. We don’t dislike the job but it is because of this reasons and not attitude problem that most of us prefer dispensing” [Referral hospital, Focus group#2].*

Surprisingly enough, there were also enthusiastic hospital pharmacists who did not see things from resource or financial gains perspective but devoted themselves for the growth of the profession. For these groups of participants, human resource was not a challenge if they were given the support from health managers, and which in turn, greatly impacted the staff’s motivation and commitment. If given the support from the management, participants considered this as their major driving force for their motivation.

*“Nowadays, there is also support from the management and this has been increased from time to time, and this is a motivating factor by itself” [Mixed hospitals, Focus group#8].*

*“So, the changes I have seen at the management is like incentives for us”* [Mixed hospitals*, Focus group#8*].

#### Social influences

Although hospital pharmacists were very much enthusiastic for new roles, these were in fact, influenced by the lack of acceptance of their role to other members of the health care team and lack of managerial support in implementing clinical pharmacy services. From the perspective of managerial support, managers overlooked clinical services but more focused on dispensing roles and that was attributed majorly to the lack of staff to take over the dispensing role. There were also participants expressed their views that managers acknowledged the importance of clinical pharmacy services and highly appreciated it but because of the staff shortage matters, those pharmacists who were working in the hospital wards were assigned to the dispensing rooms. This was more aggravated when more staffs had increasingly left their job whenever they got other better opportunities. In addition, controversies over interest also mentioned as a reason for not continually deliver this service, particularly between managers from the department and the hospital.

*“He* [the hospital manager*] is ambitious to develop the service more. However, when you come to the department of clinical pharmacy, there is a problem in the way pharmacists are looking at the service. Even, you can see that some pharmacists are not attending our morning session” [Referral hospital, Focus group#3].*

On the other hand, HCPs who were supportive and ready to accept pharmacists’ input did have some know-how about clinical pharmacy or had been exposed to some form of sensitization workshops. This was also expressed to some extent in pharmacists themselves.

*“Even other health care professionals are accepting our roles except those who don’t have the know-how. And even this is because the necessary sensitization was not given to them” [Referral hospital, Focus group#4].*

The level of acceptance was different from institution to institution. Various mentions were given for this. First, in institutions where the numbers of specialists were fewer, the input from pharmacists was taken as crucial and thus, the rate of pharmacist’s acceptance was better. However, in hospitals where there were highly experienced seniors, it was a challenge for pharmacists to recommend interventions. And, pharmacists recommended interventions were better taken up by those colleagues having the same level of seniority. However, there were also pharmacists commenting seniors had the best connections with them than others and their input was better entertained although most seniors were not that much aware of cognitive services delivered by hospital pharmacists. In addition, those HCPs who believed in team and collaborative works were the most likely candidates for promoting clinical pharmacy services.

*“We know that pharmacists working in Debre Markos and Felege Hiwot are doing better, and have better acceptance. Because their level is almost equal” [Teaching hospital, Focus group#5].*

*“With seniors, there is no problem to accept your recommendation. Actually, the main prescribing authority rests on them. The main problem is with other staffs below seniors; they need an approval from seniors. As compared to interns, the GPs* [General practitioners] *accept you better” [Referral hospital, Focus group#3].*

Participants mentioned that clinical pharmacy services were included as one of the hospital standards and had been getting the support from government policy side, and thus, no health care staff opposed the existences of these services. Notably, government’s commitment to enact on behalf of the hospital pharmacist’s impact in the health care system has been found more influential than ever, and the likelihood of accepting pharmacists extended roles to other staffs is possibly geared by the government’s pressure.

*“…So, everything rests on the government’s commitment. Our acceptance also depends on the government’s work. If the government is committed, for example, to order every health care professional to review our recommendation, like nurses, are checking the progress notes of physicians, physicians should also review the progress notes of clinical pharmacists, and give their decision as accepted or rejected. The biggest responsibility is to the government for other staffs to consume pharmacist’s input” [Referral hospital, Focus group#2].*

#### Social/professional role

Regarding medication safety activities delivered by hospital pharmacists, it was mentioned that professional compatibility was not a concern but what matters was the lack of understanding of the profession’s mission in the eye of other health care cadres. There was a considerable variation in the clinical practice of pharmacists among institutions—for example, there were institutions that praise the role of hospital pharmacists and yet, there were who had seen them as fault finders. One pharmacist commented:

*“During identifying DTPs* [drug therapy problems] *and any other problems related to medications, they are considering like we are pointing the one who is responsible for the care of the patient” [Mixed hospitals, Focus group#8].*

Whatever it is, however, the major facilitating factor for this was, role recognition by other staff members.

*“Those who understand the health benefit of clinical pharmacy services, for example, some physicians are trying to call hospital pharmacists for ward round participation, and give the recognition for clinical pharmacists as we are needed during ward round” [Mixed hospitals, Focus group#8]. “There are times when the physicians don’t start round unless the clinical pharmacist is available” [District hospital, Focus group#6].*

It was mentioned that the hospital standards currently ratified by the government well advocated the integration of pharmacists in care teams. However, few pharmacists believed there were a lack of distinction between technical and clinical services and role duties for pharmacists from the government itself.

*“The government did not see the distinction between technicians, pharmacists, and clinical pharmacists” [District hospital, Focus group#7].*

Although there existed some level of recognition from various sides, yet there had been a lack of awareness regarding the role of hospital pharmacists in medication safety activities at the level of health bureau, regional or federal level.

*“…there are staffs who are not aware of the role of clinical pharmacists. There are staffs who ask us what we are doing in the ward, on the other hand, there are who eagerly want us, and even among these, there do have various perceptions of the profession” [Referral hospital, Focus group#2].*

With regard to social/professional role, there had been numerous unfinished assignments that due attention, according to the participants. Awareness campaigns should be devised, and a well-designed job description should have been in place. Because of the lack of job description best suited for clinical activities, participants felt that there seemed an overlap of activities and also, other HCPs perceived as if their role was taken. Few participants commented how other staffs, specifically physicians were looking at them; they stressed that their therapy recommendation was not usually entertained by the physicians, and the physicians hesitate to accept their extended role.

Regardless of financial gains and acceptance, most hospital pharmacists were positive towards the future of the profession.

*“We are taking the challenges as challenges, and we are thinking the future might be brighter. We don’t know what will happen and in that sense, we are trying our best” [District hospital*, *Focus group#6].*

“*…we are working for the benefit of the profession, not for us, we are paying our life, and we are wishing only the best future” [District hospital*, *Focus group#6].“…we are working expecting the future might be bright” [District hospital*, *Focus group#6].*

## Discussion

To the best of our knowledge, this was the first study to apply the TDF to categorize the barriers and facilitators to hospital pharmacists’ engagement in medication safety activities. The present study identified a wide range of factors that may influence the uptake of medication safety interventions delivered by hospital pharmacists. Overwhelmingly, hospital pharmacists identified more barriers than facilitators in delivering clinical services. Derived from the TDF, the factors identified in this study were clustered into six domains: ‘Knowledge’, ‘Skills’, ‘Environmental context and resources’, ‘Motivations and goals’, ‘Social influences’ and ‘Social/professional role’. In comparison with other studies using the TDF framework, the domains ‘Knowledge’, ‘Skills’, ‘Environmental context and resources’, ‘Social influences’ were identified as vital areas which could be targeted in the implementation of medication safety programs [32, 33, 35], although other issues such as, ‘Motivation and goals’ [32, 33] and ‘Social/professional roles’ [32] were also equally important. Outside the TDF, some of our findings were consistent with a previous study exploring the factors affecting the implementation of clinical pharmacy performance indicators, including medication reconciliation [38]. Minard et al. [38] reported that the challenges surrounding hospital pharmacists’ implementation of these indicators comprised of documentation challenges, work burden, environmental constraints and competing priorities. Using a theory-based approach, the present study uncovers additional relevant barriers—for example, the lack of knowledge and skills necessary for the execution of clinical services and poor acceptance of pharmacists’ recommendation. On the other hand, environmental constraints identified in the current study were prominent, and there was none which was mentioned as facilitator in the context of resource issues. Most importantly, although all participants frequently and consistently reported the ‘Environmental context and resources’ domain without variation in their views, it was found that there existed some important interlinks with the domains judged to be relevant. For example, as a consequence of human resource deficits, managers reinforced hospital pharmacists to take over the dispensing role (‘Motivation and goals’), and because of the absence of duty and weekend programs, hospital pharmacists perceived this as a lack of government funding and support, which in turn, was a result of the lack of recognition and acceptance of these extended roles (‘Social/professional role’). Duncan et al. [32] explained the interactive nature across the TDF domains and highlighted the importance of considering theoretical links between domains as far as interrelationships between domains exist.

Unlike the environmental constraints, the barriers and facilitators that were reported by hospital pharmacists showed inter-institutional and -individual variations in the remainder of domains. While the analysis of the interview data indicated major differences in individual thoughts related to hospital pharmacists’ knowledge, skills, and social/professional role as well as their motivation and goals, inter-institutional variation mainly appeared in the social influence domain. Particularly, hospital pharmacists working in district hospitals clearly indicated their interventions were better entertained and accepted by other health care members, and there was an increasing demand for these services—for example, expressed in the number of telephone inquiries and consultations received in these hospitals. Previous studies demonstrating pharmacist provided therapy recommendation in care teams have reported positive clinical and economic outcomes, and these have been associated with high acceptance rates [39–41]. For instance, when pharmacists participate in ward rounds, they could able to cut two-thirds of preventable ADEs with acceptance rate as high as 99% [40]. Another recent study [42] has also shown a high acceptance rate of pharmacist-provided services associated with medication reconciliation as compared with other clinical services, such as those related to medication indication, efficacy, and therapeutic drug monitoring. Given the positive impact of pharmacist-led medication reconciliation services [22, 23], and the evidence that these services have shown better acceptance [42], it is our opinion that pharmacists’ clinical services in the studied hospitals, mainly those above the district level, might be well utilized if they could able to implement medication reconciliation services.

Apart from the challenges encountered with regard to knowledge and skill deficits (e.g. lack of supportive skills such as blood pressure measurement, and knowledge about rare diseases/diagnosis)—participants associated this with the challenges in the currently designed curricula, hospital pharmacist’s roles in medication safety were poorly understood in the medical community. Particularly, product-oriented pharmacists’ awareness, and the lack thereof, was predominantly affected the extended roles implemented by ward-based pharmacists. This finding is consistent with a study that has shown pharmacists’ self-perception as barriers to their extended roles [43]. Outside pharmacists, participants highlighted that other HCPs recognition of pharmacist’s roles in medication safety activities was limited; however, a recent local study reported that a large proportion of HCPs had a positive attitude towards clinical pharmacy services but the extent of the available service was below their expectation [44]. The present study has also identified that, in the eyes of health managers, dispensing was thought to be a core business and thus, hospital pharmacists were reinforced for other competing priorities. There were no remuneration schemes or incentives arranged for pharmacist’s cognitive services. As a result, many pharmacists preferred dispensing to clinical services. A previous national study has also shown that close to two-thirds of pharmacists delivering clinical pharmacy services are dissatisfied with their job, and this is mainly due to unattractive incentive packages [25].

All participants expressed a desire for further trainings and certifications to target their knowledge and skills gaps; this was also a motivating factor for delivering these services. To target other domains (e.g. ‘Social/professional role’, ‘Social influence’), awareness creation campaigns targeting the whole medical community (including the management, other pharmacists, and HCPs) may possibly facilitate the uptake of pharmacist’s cognitive services. In addition, government recognition and supervision of hospital pharmacists’ clinical services have been cited as a main driving factor, and participants perceived these services should not have been confined to few settings and national endorsement of these services have been found to be necessary. Bilal et al. [45] have also confirmed that Ethiopian graduate pharmacists are very much enthusiastic to promote clinical pharmacy service but the challenge is the minimal effort made at the level of institutions.

This study has several strengths and limitations. Applying the TDF approach, we have for the first time identified a range of barriers, as well as facilitators in relation to hospital pharmacists’ engagement in medication safety activities. As we employed focus group discussions for data collection, the data generated was possibly rich [46], and also, the interview guide was structured across the TDF domains that could able to elicit as many factors as possible, although this renders prioritization of domains for intervention development difficult [47]. However, we adopted the relevance criteria utilized by previous studies for prioritizing domains of potential interventional targets [32, 34]. One important challenge in relation to coding statements into the theoretical domains was the existence of overlaps between domains. In this instance, it might be difficult to determine the origin of barrier and facilitator and prioritize interventions [48]. Fortunately, in the current study, the domains that have been found with some interrelationships were included in the priority list of behaviours for possible intervention.

In contrast to other studies which also judged beliefs about capabilities [49], beliefs about consequences [33, 49], memory/attention and decision processes [32, 35, 49] as relevant domains for a successful medication safety intervention, these domains in our study were described infrequently (‘Memory/attention and decision processes’) and varied little (‘Beliefs about capabilities’), and participants were confident enough in the positive impact of clinical pharmacy services (‘Beliefs about consequences’). Although participants consistently reported challenges to the service delivery but in their accounts, we understood that was meant barriers encountered in the whole process and not attributed to their incapability in carrying out medication review and reconciliation, for example. Additionally, an important point worth discussing is regarding the targeted behaviour (i.e. medication safety activities delivered by hospital pharmacists) that we would like to intervene have certain unique features as compared to other studies. While other studies focused on some specifically targeted behaviours (e.g. prescribing behaviour [32, 35], prescribing and dispensing behaviour [35] and ADE reporting [33]), our study included a range of bundled interventions, also termed as clinical pharmacy services. A core sets of eight clinical pharmacy performance indicators have been established [38], including admission and discharge medication reconciliation. However, the issue of medication reconciliation was new to the local setting, and we intended to ask our interviewees from the broader perspective rather than as an isolated element, and interview questions had been designed, accordingly. Hospital pharmacists expressed their beliefs from the broader array of these services, and beliefs for each of the afro mentioned domains should have been thus, viewed from that angle.

Another study’s limitation was that it involved a homogeneous group of participants; that is, only pharmacists who taught in the newly designed patient-oriented curricula were included. It did not take the thoughts from the perspective of product-oriented pharmacists. However, pharmacists were sampled from eight hospitals of various levels (district, general and tertiary), and this mix could possibly enhance transferability of findings to other settings.

## Conclusion

This is the first study to investigate the potential barriers and facilitators to implementing evidence-based medication safety activities delivered by hospital pharmacists using the TDF and is an initial step necessary for informing theory-based interventions to target these barriers. The current study sheds light on hospital pharmacists’ perceptions of their clinical services, including medication reconciliation, in settings where resources are limited. The majority of the participants were very much enthusiastic for their extended roles and were positive towards the future of the profession; however, there were a number of factors likely to influence their behavior in the clinical practice of pharmacists. The multifaceted behavioural interventions surrounding hospital pharmacist’s engagement in medication safety activities were predominantly related to six theoretical domains: ‘Knowledge’, ‘Skills’, ‘Environmental context and resources’, ‘Motivations and goals’, ‘Social influences’ and ‘Social/professional role’. There existed considerable interrelationships between domains that were perceived to influence hospital pharmacists’ behaviours, and this might assist in designing behaviour change interventions that target common behavioural domains.

## Additional files

Additional file 1: TDF-based interview topic guide. (DOCX 13 kb)

Additional file 2: Coding guide. Description of 12 theoretical domains from TDF. (DOCX 13 kb)

## Abbreviations

ADE: Adverse drug event
BPharm: Bachelor of Pharmacy
FGD: Focus group discussion
FMHACA: Food, Medicine and Health Care Administration and Control Authority
GP: General practitioner
TDF: Theoretical Domains Framework
USA: United Sates of America
