# Supplementary material for: Barriers and facilitators to hospital pharmacists’ engagement in medication safety activities: a qualitative study using the theoretical domains framework
Source: J Pharm Policy Pract. 2018 Jan 23;11:2. doi: 10.1186/s40545-018-0129-y (PMC5778635; doi:10.1186/s40545-018-0129-y)
Supplement: Supplementary file 1 — TDF-based interview topic guide. (DOCX 13 kb) [file 40545_2018_129_MOESM1_ESM.docx]

**Additional file 1:** TDF-based interview topic guide

| Domains | Interview questions |
| --- | --- |
| Knowledge | Are there any hospital guidelines for pharmacists to deliver clinical pharmacy services?  What do you think the level of evidence is for these guidelines?  What do you know about medication reconciliation and review?  Can you describe pharmacists’ roles in medication safety activities? |
| Skills | Do you know how to deliver clinical pharmacy services?  Do you know how to deliver medication reconicilaition and review servies?  Is identification of medication related problems difficult for you?  Have you atteneded in-serivce training to deliver clinical pharmacy services? |
| Social/professional role | Is doing medication reconicilation and review compatible with your professional role?  Who is responsible for these services at your hospital?  Do you think hospital guidelines supports your professional roles as a pharmaceutical care practitioners? |
| Beliefs about capabilties | How easy or difficult do you find performing clinical pharmacy activities ?  What problems have you encountered?  How capable are you in performing medication reconciliation and review?  How confident are you that you can do these services despite difficulties?  How comfortable do you feel to undertake these services? |
| Beliefs about consequences | What are the likely positive/negative outcomes of reporting/communicating medication related problems?  What are the costs of delivering medication reconiciliation and review and what are the costs of the consequences of these services?  Are you concerned if these services are not provided at your hospital?  Do benefits of doing these services outweigh the costs?  Does the evidence suggests that doing these services are beneficial? |
| Motivation and goals | How motivated are you to deliver medication reconciliation and review?  Are there incentives to provide these services?  Do you have any other hospital activity that hinders these services? |
| Memory, attention and decision processes | Will you consider providing medication reconcilation and review services? If so, how frequently would you undertake this activity?  How much priority have you given to these services? |
| Enviromental context and resources | To what extent do physical factors or resources facilitate or hinder to deliver medication reconicilation/review?  Are there competing tasks and time constraints?  Are the necessary resources available to undertake these services?  Do these services have advantages compared with the standard care?  Do government and local authorties provide sufficient support for these services? |
| Social influences | Are clinical pharmacy services in the hospital well acknowledged by other healthcare professionals?  Do hospital managers acknowledge your role?  Is there any obstruction to these activities in your hospital?  Have you observed others doing providing these clinical services? |
| Emotion | What things worry you the most in providing medication reconcilation/review services?  To what extent do emotional factors facilitate or hinder these serivces? |
| Behavioural regulation | Have you received feeedback from other healthcare professionals regarding these services?  What initial steps are needed to deliver these services? |
| Nature of the behaviours | What do you currently do?  How long will changes going to take?  Are there any systems in place for sustainable long term changes? |
